# Genetic Variation Stimulated by Epigenetic Modification

**DOI:** 10.1371/journal.pone.0004075

**Published:** 2008-12-30

**Authors:** W. Jason Cummings, David W. Bednarski, Nancy Maizels

**Affiliations:** 1 Department of Immunology, University of Washington School of Medicine, Seattle, Washington, United States of America; 2 Department of Biochemistry, University of Washington School of Medicine, Seattle, Washington, United States of America; Brandeis University, United States of America

## Abstract

Homologous recombination is essential for maintaining genomic integrity. A common repair mechanism, it uses a homologous or homeologous donor as a template for repair of a damaged target gene. Such repair must be regulated, both to identify appropriate donors for repair, and to avoid excess or inappropriate recombination. We show that modifications of donor chromatin structure can promote homology-directed repair. These experiments demonstrate that either the activator VP16 or the histone chaperone, HIRA, accelerated gene conversion approximately 10-fold when tethered within the donor array for Ig gene conversion in the chicken B cell line DT40. VP16 greatly increased levels of acetylated histones H3 and H4, while tethered HIRA did not affect histone acetylation, but caused an increase in local nucleosome density and levels of histone H3.3. Thus, epigenetic modification can stimulate genetic variation. The evidence that distinct activating modifications can promote similar functional outcomes suggests that a variety of chromatin changes may regulate homologous recombination, and that disregulation of epigenetic marks may have deleterious genetic consequences.

## Introduction

Homologous recombination depends upon a DNA donor molecule to serve as a template for correction of a damaged recipient [1], [2]. Homologous recombination is a critical pathway for error-free restoration of broken DNA, but it can also lead to mutagenesis and chromosomal rearrangements. templated by a homolog rather than a sister chromatid can lead to loss of heterozygosity associated with both cancer and aging; and homologous recombination between nonallelic repeated sequences can cause genomic instability and human genetic disease [3], [4], [5], [6].

Chromatin status contributes to regulation of homologous recombination. Dynamic changes in chromatin structure occur at a site of DNA damage, and are important for maintaining broken DNA ends in close proximity [7], [8], [9], [10], [11]. In addition, we recently showed that local repressive modifications at donor chromatin can diminish homologous recombination [12]. This raised the possibility that activating modifications might stimulate recombination. We have now tested this, by asking if gene conversion can be promoted by local recruitment to the donors of factors associated with activation of chromatin. We assayed the effects of two distinct regulators, VP16 and HIRA. VP16 is a potent and well-characterized transactivator derived from herpesvirus, which has been associated with the relaxation of chromatin and and is known to interact with chromatin remodeling [13], [14] and histone acetyltransferase complexes [15], [16]. HIRA is a histone chaperone capable of nucleosome assembly and deposition outside of S-phase [17], [18], with a role in deposition of the histone variant H3.3 [17], [19].

Tethered VP16 or HIRA caused the level of gene conversion to increase by approximately an order of magnitude (8.4-fold and 11.0-fold, respectively). While these two tethered factors had similar functional outcomes, the localized changes in chromatin structure that they produced were quite distinct. Tethered VP16 greatly increased levels of acetylated histones, a mark associated with permissive DNA structure. Tethered HIRA did not alter local histone acetylation, but increased nucleosome deposition and caused local ordering of chromatin structure. Thus, different pathways of modification, and therefore different chromatin states, can achieve the same end: promotion of homologous recombination. Epigenetic regulation may be an important mechanism of both preserving and modifying genomic structure.

## Results

### Tethered VP16 accelerates gene conversion

**Figure 1:**
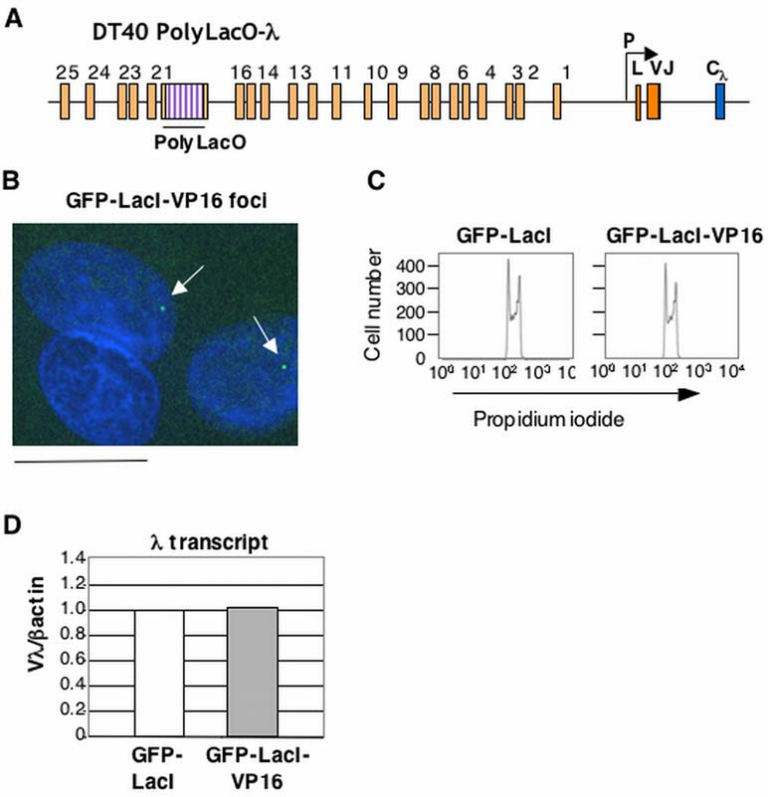
Tethering of GFP-LacI-VP16 to the ψVλ Array in DT40 PolyLacO-λR. (A) Schematic diagram of the rearranged chicken Igλ locus in DT40, with polymerized lactose operator (PolyLacO) inserted between ψVλ17–20. Promoter, P; leader, L; rearranged variable region, Vλ–Jλ; constant region, Cλ. (B) Fluorescent image of DT40 PolyLacO-λ GFP-LacI-VP16 transfectants. Nuclei were counterstained with DAPI (blue). Arrows indicate GFP-LacI-VP16 bound to Igλ. Bar, 15 µm. (C) Cell cycle profiles of DT40 PolyLacO-λ GFP-LacI and DT40 PolyLacO-λ GFP-LacI-VP16 transfectants. (D) Quantitation of RT-PCR comparison of Vλ transcript levels in DT40 PolyLacO-λ GFP-LacI and DT40 PolyLacO-λ GFP-LacI-VP16 transfectants. Values represent the average of at least 2 amplifications that were normalized to the average value for DT40 PolyLacO-λ GFP-LacI.

To determine the effects of chromatin structure on gene conversion, we have taken advantage of the powerful physiological model provided by the chicken B cell line, DT40. In the DT40 cell line, which derives from a B cell lymphoma, the Ig heavy (IgH) and light (Igλ) chain variable (V) regions constitutively diversify by gene conversion. Gene conversion is templated by an array of homeologous upstream pseudo-V (ψV) regions (Figure 1A), which lack promoters and are nonfunctional. The ψVλ donors are enriched for acetylated histones H3 and H4 (AcH3 and AcH4), although they are not transcribed [12].

To ask if further enrichment of AcH3 and AcH4 could be achieved, or could affect recombination, we used the DT40 PolyLacO-λ cell line constructed by our laboratory. In this cell line, polymerized lactose operator (PolyLacO) has been inserted into the ψVλ array between ψVλ17–ψVλ20, 17 kb upstream of the expressed Vλ gene (Figure 1A; [12]. This allows us to assay the effects of tethered regulatory factors expressed as fusions with lactose repressor. We generated DT40 PolyLacO-λ transfectants stably expressing the activation domain of VP16 fused to GFP-LacI, GFP-LacI-VP16. A single green spot was readily imaged within the nuclei of the DT40 PolyLacO-λ GFP-LacI-VP16 transfectants, providing evidence of GFP-LacI-VP16 expression and binding to PolyLacO (Figure 1B). The cell cycle profile (Figure 1C) and levels of λ transcription (Figure 1D) were unaltered relative to control DT40 PolyLacO-λ GFP-LacI transfectants.

**Figure 2:**
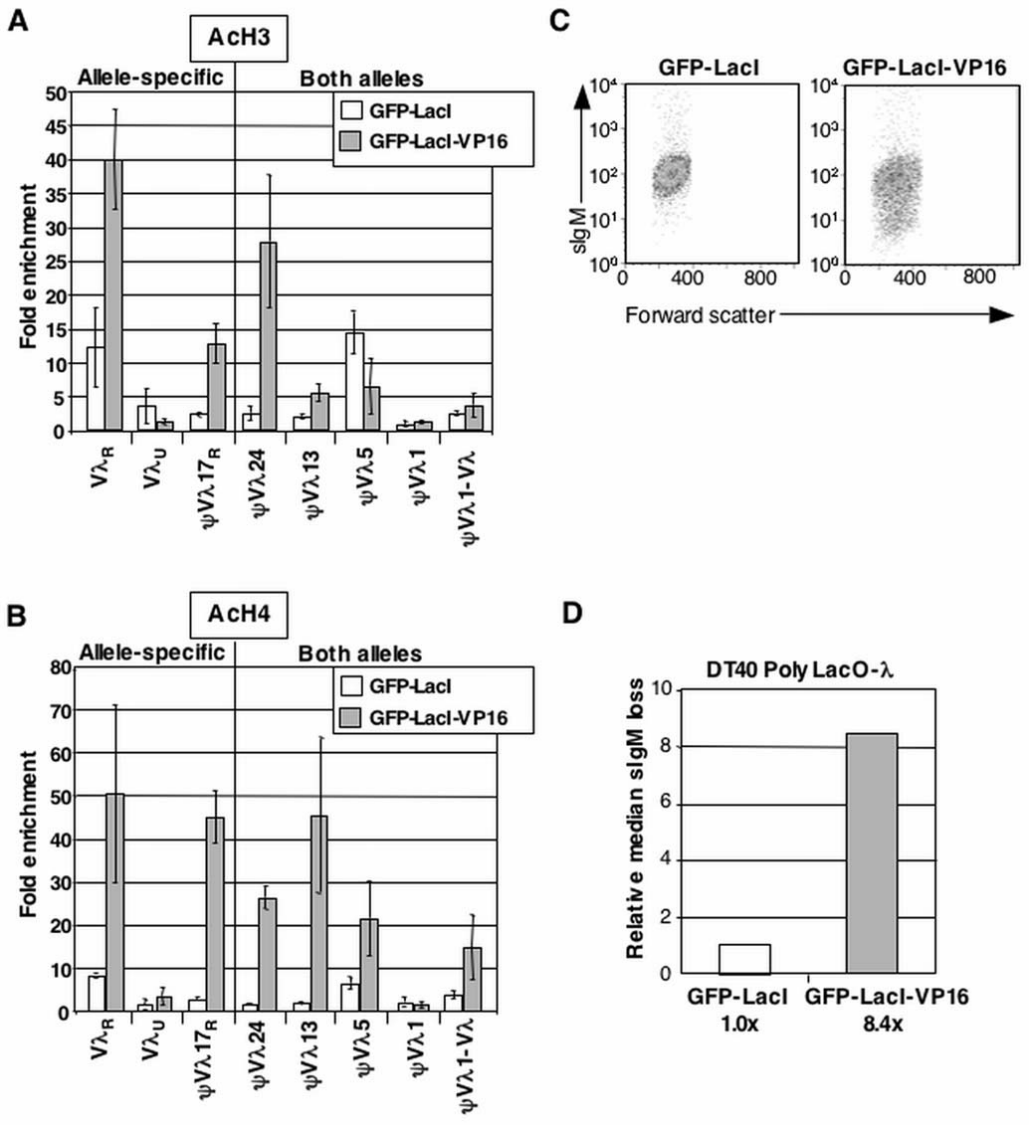
Tethered VP16 Increases AcH3 and AcH4 Levels and Accelerates Ig Gene Diversification. (A) Quantitation of enrichment of acetylated H3 (AcH3) at Igλ in DT40 PolyLacO-λ GFP-LacI and DT40 PolyLacO-λ GFP-LacI-VP16 transfectants. Sites interrogated are shown below. Enrichment assayed by ChIP is expressed relative to the total DNA input control, ±standard deviation of three or more separate amplifications of increasing amounts of template DNA. (B) Quantitation of enrichment of acetylated H4 (AcH4) at Igλ in DT40 PolyLacO-λ GFP-LacI and DT40 PolyLacO-λ GFP-LacI-VP16 transfectants, expressed relative to DNA input control. Notations as in Panel A. (C) Flow cytometry of individual expanded clones of DT40 PolyLacO-λ GFP-LacI and DT40 PolyLacO-λ GFP-LacI-VP16 transfectants, stained with anti-IgM. (D) Median sIgM loss in panels of independent DT40 PolyLacO-λ GFP-LacI-VP16 transfectants (n = 64), normalized to DT40 PolyLacO-Vλ GFP-LacI transfectants (n = 27). The figure shows combined data from at least two independent transfectants for each construct. The fold increase relative to control DT40 PolyLacO-Vλ GFP-LacI transfectants is shown below.

We assayed histone acetylation in PolyLacO-λ GFP-LacI-VP16 and control DT40 PolyLacO-λ GFP-LacI cells by chromatin immunoprecipitation (ChIP). There are two λ light chain alleles in DT40 cells, one rearranged and actively expressing λ light chain (λ_R_), and the other unrearranged and not transcribed (λ_U_). Allele-specific chromatin modifications were assayed at the rearranged allele of Vλ (Vλ_R_); the unrearranged allele of Vλ (Vλ_U_); and ψVλ17 (ψVλ17_R_), where sequence polymorphism was created by insertion of PolyLacO-λ, providing the only site in the ψVλ array at which the rearranged and unrearranged alleles can be distinguished by use of specific PCR primers. Modifications on both alleles were assayed by amplification of five sites, including the region between ψVλ1 and the Vλ gene (ψV1–Vλ); ψVλ1; ψVλ5; ψVλ13; ψVλ24. Strikingly, tethered VP16 increased levels of both AcH3 (Figure 2A) and AcH4 (Figure 2B); while tethered GFP-LacI did not have this effect. Enrichment of AcH3 was 40-fold relative to input at the transcribed Vλ_R_ allele, and 10-fold at the allele-specific site, ψVλ17. There was essentially no enrichment at the nontranscribed Vλ_U_ allele (Figure 2A, left), indicating that the activity of VP16 was specifically targeted to the PolyLacO-containing allele. AcH4 also displayed allele-specific enrichment, in the range of 45- to 50-fold at Vλ_R_ and ψVλ17; and 20-fold at most upstream sites in the ψVλ array. Very little enrichment was observed at the most downstream element, ψVλ1. This site similarly shows very little enrichment in AcH3 or AcH4 in the parental DT40 line [12],

Comparison of enrichment of AcH3 and AcH4 in DT40 PolyLacO-λ cells in which either GFP or VP16 was tethered to the ψVλ array showed that tethered VP16 caused a dramatic increase in local histone acetylation at almost all sites interrogated. An exception was ψVλ5, where tethered VP16 diminished AcH3 levels. This site is notable for its naturally high levels of AcH3 and AcH4 in the parental DT40 cell line [12]. As with AcH3, we did not observe any enrichment of AcH4 at the nontranscribed Vλ_U_ allele control (Figure 2B). The dramatic enrichment of both AcH3 and AcH4 at the rearranged and expressed allele Vλ_R_ is especially notable because tethered VP16 did not cause an increase in Vλ transcript levels (Figure 1D).

We used the sIgM loss variant assay to ask if tethered VP16 altered the clonal rate of Vλ sequence diversification. This fluctuation assay measures the fraction of cells that no longer express structurally intact sIgM, and thus scores mutation events resulting from gene conversion, point mutation, insertion or deletion [12], [20], [21]. While gene conversion is the predominant pathway of Ig gene diversification in chicken B cells, if gene conversion is impaired (for example by the absence of essential recombination factors [20], [22], [23], [24], [25], [26], other repair outcomes, particularly point mutations, will predominate. Thus diversification is monitored best by this loss of function assay, rather than an sIgM^−^ to sIgM^+^ gain of function assay, which scores only gene conversion [27] and is sensitive to variations in reversion frequencies at different sites within Vλ [28].

Independent clonal derivatives of DT40 PolyLacO-λ GFP-LacI and DT40 PolyLacO-λ GFP-LacI-VP16 transfectants were established by limiting dilution cloning of sIgM^+^ cells, and following 4 weeks of culture the fraction of sIgM^−^ cells in each population was determined by flow cytometry of cells stained with anti-IgM antibody. A considerable fraction of sIgM^−^ cells was evident in most clonal populations of DT40 PolyLacO-λ GFP-LacI-VP16 transfectants relative to DT40 PolyLacO-λ GFP-LacI transfectants (e.g. Figure 2C). Comparison of median percentages of sIgM^−^ cells showed that DT40 PolyLacO-λ GFP-LacI-VP16 transfectants exhibited an 8.4-fold increase in clonal diversification rate relative to DT40 PolyLacO-λ GFP-LacI control cells (Figure 2D).

### Histone H3.3 is enriched at the ψVλ donor genes

**Figure 3:**
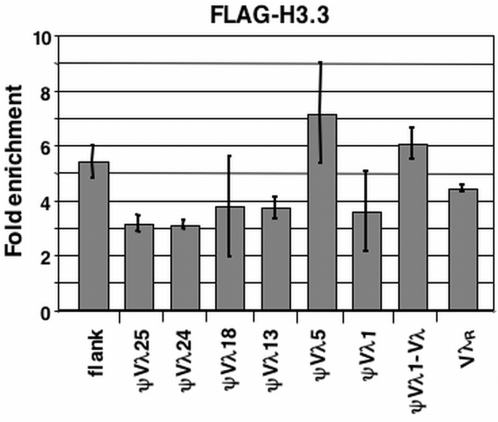
The H3.3 Histone Variant is Enriched at the DT40 Igλ Locus. Summary of a representative chromatin immunoprecipitation experiment, assaying enrichment of the H3.3 histone variant. Sites interrogated were: a region approximately 1 kb upstream of the ψVλ array (flank); the region between ψVλ1 and Vλ; ψVλ1; ψVλ5; ψVλ13; ψVλ18; ψVλ24; ψVλ25; and the rearranged Vλ_R_ allele. Bars indicate standard deviation of at least three separate amplifications.

One histone variant associated with chromatin activation and deposited independently of replication is H3.3, the principal H3-like variant to contain activation-associated postranslational modifications [29], [30]. As the presence of H3.3 could provide another mechanism to activate donor chromatin for recombination, we asked if H3.3 was enriched at the ψVλ array. To distinguish H3 from H3.3 by ChIP, we generated DT40 derivatives that stably express FLAG-tagged human H3.3 (H3.3-FLAG; [31] and characterized H3.3 deposition by ChIP with an anti-FLAG antibody. H3.3-FLAG was 4-fold enriched at the expressed Vλ_R_ gene relative to input DNA, and considerably enriched throughout the ψVλ array (Figure 3). Because levels of H3.3-FLAG expression may vary among different transfectants, we normalized levels at the light chain locus relative to levels at the ovalbumin (Ova) gene, which is not expressed in B cells and was devoid of H3.3. Enrichment of H3.3 within the ψVλ array does not simply represent a graded spreading of this variant from the transcribed Vλ_R_ gene to sites upstream, as sites near Vλ_R_ did not consistently display higher levels of enrichment than more distant sites. This non-uniform chromatin structure suggests that *cis*-elements, particularly in the ψVλ5 region, may be especially important for regulation of expression and/or diversification.

### Tethered HIRA promotes gene conversion

**Figure 4:**
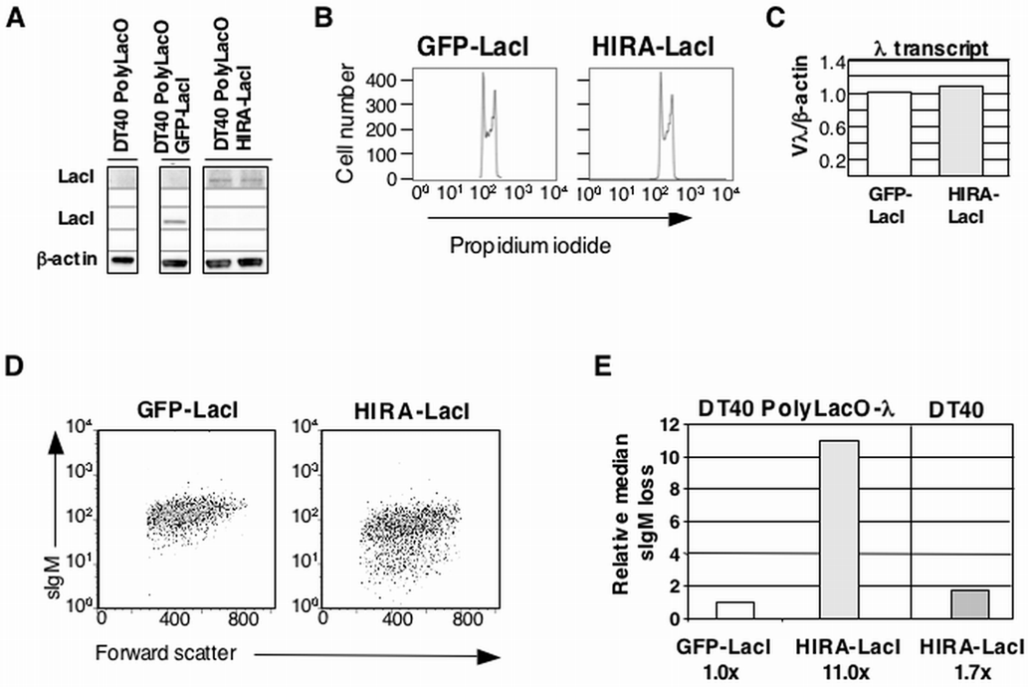
Tethered HIRA Accelerates Ig Gene Diversification. (A) Western blot analysis of expression of GFP-LacI or HIRA-LacI in indicated transfectants. Two individual clones are shown for HIRA-LacI expression. Assay was performed by blotting with anti-LacI antibodies (for detection of GFP-LacI and HIRA-LacI), relative to β-actin control. (B) Cell cycle profile of DT40 PolyLacO-λ GFP-LacI and DT40 PolyLacO-λ HIRA-LacI transfectants. (C) Quantitation of RT-PCR comparison of Vλ transcript levels in DT40 PolyLacO-λ GFP-LacI and DT40 PolyLacO-λ HIRA-LacI transfectants. Values represent the average of at least 2 amplifications that were normalized to the average value for DT40 PolyLacO-λ GFP-LacI. (D) Flow cytometry of individual expanded clones in representative populations of DT40 PolyLacO-λ GFP-LacI and DT40 PolyLacO-λ HIRA-LacI transfectants, stained with anti-IgM. (E) Median sIgM loss in panels of independent DT40 PolyLacO-λ HIRA-LacI (n = 48), and DT40 HIRA transfectants (n = 24), normalized to DT40 PolyLacO-λ GFP-LacI transfectants (n = 24). The figure shows combined data from at least two independent transfectants for each construct. The fold increase relative to control DT40 PolyLacO-λ GFP-LacI transfectants is shown below.

Enrichment of H3.3 at the ψVλ donors raised the possibility that this variant histone may be important for efficient homologous recombination. The histone chaperone HIRA can promote assembly of nucleosomes containing histone H3.3 [17], [19], suggesting that tethered HIRA might accelerate Ig gene diversification, analogous to tethered VP16. To test this, we generated stable DT40 PolyLacO-λ HIRA-LacI transfectants, verified expression of the protein by Western blotting (Figure 4A), and showed that tethered HIRA did not affect cell cycle profile (Figure 4B) or λ transcript levels (Figure 4C).

To ask if tethered HIRA affects the clonal rate of Ig gene diversification, we established independent clonal populations of DT40 PolyLacO-λ HIRA-LacI transfectants by limiting dilution cloning of sIgM^+^ cells, and following 4 weeks of culture the fraction of sIgM^−^ cells in each population was determined. Flow cytometry showed a considerable fraction of sIgM^−^ cells in most HIRA-LacI transfectants, but not in GFP-LacI control transfectants (e.g. Figure 4D). Comparison of median percentages of sIgM^−^ cells showed that DT40 PolyLacO-λ HIRA-LacI cells exhibited an 11.0-fold increase in diversification relative to DT40 PolyLacO-λ GFP-LacI controls (Figure 4E). This did not simply reflect overexpression of HIRA, as only a modest increase in the rate of clonal diversification (1.7-fold) was evident in transfectants of DT40 lacking a PolyLacO element but stably expressing HIRA-LacI (Figure 4E).

### Tethered VP16 and tethered HIRA promote gene conversion

Sequence analysis of Vλ regions amplified by single cell PCR from cells expressing tethered VP16 or HIRA showed that most diversification was by gene conversion (Supplementary Figures S1 and S2). Table 1 summarizes mutational events. The great majority of sequence changes in both DT40 PolyLacO-λ GFP-LacI-VP16 transfectants and DT40 PolyLacO-λ_R_ HIRA-LacI transfectants were due to gene conversion (82% and 87%, respectively), comparable to levels of gene conversion documented in DT40 PolyLacO-λ GFP-LacI transfectants (80%; Cummings et al. 2007). However, the fraction of gene conversion events containing only a single substitution per tract was considerably higher in DT40 PolyLacO-λ GFP-LacI-VP16 transfectants and DT40 PolyLacO-λ_R_ HIRA-LacI transfectants (37% and 24%, respectively) than in DT40 PolyLacO-λ_R_ GFP-LacI transfectants (3%). Thus, while essentially all gene conversion tracts in the GFP-LacI control cells contained at least two mutations, acceleration of diversification by either tethered VP16 or HIRA was accompanied by a diminished number of nucleotide substitutions per tract.

**Table 1:** Effect of Tethered HIRA and VP16 on Gene Conversion.

			GFP-LacI-VP16	HIRA-LacI	GFP-LacI
**Gene Conversion**			**82%**	**87%**	**80%**
	tracts with				
		≥2 substitutions	63%	76%	97%
		1 substitution	37%	24%	3%
**Point mutation**			**7%**	**11%**	**20%**
**Insertion**			**9%**	**2%**	**0%**
**Deletion**			**2%**	**0%**	**0%**

Summary of sequences of Vλ regions carrying unique mutations from DT40 PolyLacO-λ GFP-LacI-VP16 (n = 92), and DT40 PolyLacO-λ HIRA-LacI (n = 137) transfectants. Data derived from at least two independent transfections. DT40 PolyLacO-λ GFP-LacI {Cummings, 2007 #776} is included for comparison.

### Tethered HIRA causes a local increase in histone deposition and nucleosome density

**Figure 5:**
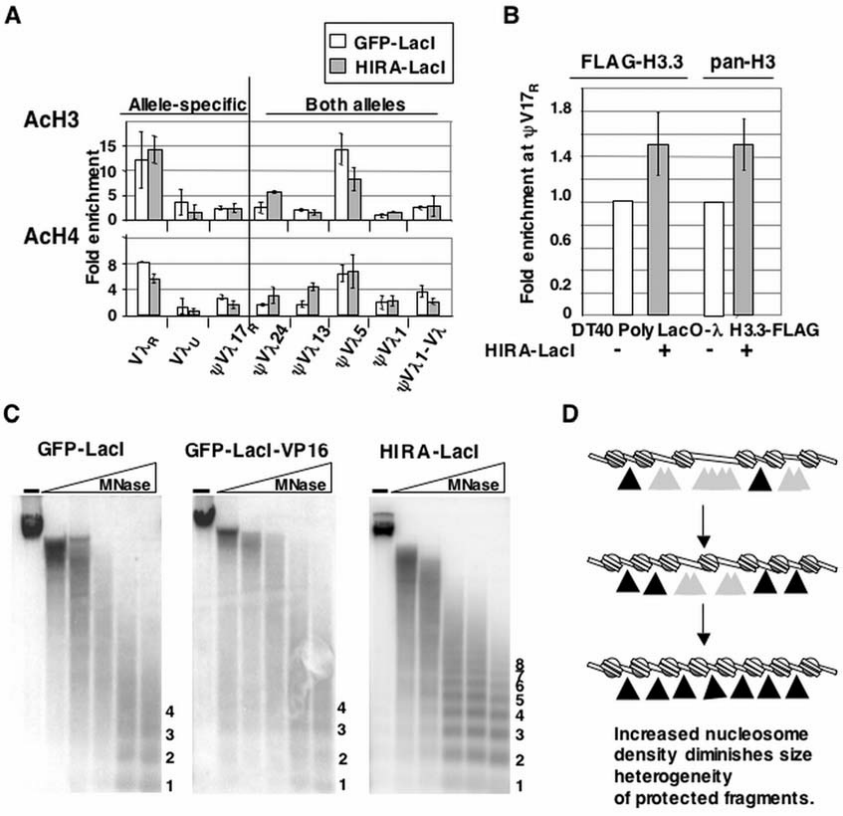
Tethered HIRA Enriches Local Levels of H3.3 and Alters Local Chromatin Structure. (A) Quantitation of enrichment of AcH3 (above) and AcH4 (below) at Igλ in DT40 PolyLacO-λ GFP-LacI and DT40 PolyLacO-λ HIRA-LacI transfectants. Enrichment assayed by ChIP is expressed relative to the total DNA input control, ±standard deviation of three or more separate amplifications of increasing amounts of template DNA. (B) Quantitation of ChIP analysis of enrichment of H3.3 (FLAG-H3.3) and pan H3 at ψVλ17_R_ in DT40 PolyLacO-λ and DT40 PolyLacO-λ HIRA-LacI cells expressing H3.3-FLAG, normalized to enrichment in DT40 PolyLacO-λ. Bars are shown ±standard deviation of four separate amplifications of increasing amounts of template DNA. (C) Southern blot analysis of the PolyLacO region in DT40 PolyLacO-λ GFP-LacI, DT40 PolyLacO-λ GFP-LacI-VP16 and DT40 PolyLacO-λ HIRA-LacI transfectants, following digestion of chromatin with micrococcal nuclease (MNase). No Mnase, (-). Numbers at the right denote nucleosome multimers. (D) Model of how elevated histone deposition could diminish size heterogeneity of protected nucleosome fragments. A defined region of DNA experiences nucleosome loading at increased density. Grey arrowheads indicate a range of cleavable positions, which will result in size heterogeneity of the protected fragments; dark arrowheads indicate restricted range of cleavable sites.

ChIP analysis showed that, in contrast to VP16, tethered HIRA did not affect AcH3 and AcH4 levels, relative to a GFP-LacI control (Figure 5A). To ask if tethered HIRA increased H3.3 levels, we generated DT40 PolyLacO-λ FLAG-H3.3 HIRA-LacI transfectants, and then determined enrichment of FLAG-H3.3 at ψVλ17_R_, where a polymorphism enables allele-specific analysis of chromatin. Those experiments showed a modest (1.4-fold) enrichment of FLAG-H3.3 relative to a control line (Figure 5B), reproducible in an independent HIRA-LacI transfectant (not shown). Moreover, a comparable enrichment was evident when pan-H3 antibodies were used for ChIP (which detect H3 and other variants, including H3.3). Thus, tethered HIRA appeared to cause a net local enrichment of histones.

To probe nucleosome loading, nuclei were harvested from DT40 PolyLacO-λ GFP-LacI, GFP-LacI-VP16 and HIRA-LacI transfectants and treated with varied amounts of micrococcal nuclease (MNase), which attacks the linker regions between nucleosomes to produce a characteristic ladder. Genomic DNA was then purified, resolved and blotted, and the PolyLacO region probed by hybridization. The MNase digestion patterns of DT40 PolyLacO-λ GFP-LacI and DT40 PolyLacO-λ GFP-LacI-VP16 cells were similar to each other (Figure 5C, left, middle) and to the parental DT40 PolyLacO-λ line (data not shown). Digestion produced a ladder of fragments (labeled 1–4 in the figure), which appear as blurred rather than sharp bands, reflecting heterogeneity in size of nucleosome multimers produced upon partial digestion. In contrast, MNase digestion of chromatin from DT40 PolyLacO-λ HIRA-LacI cells revealed a distinctive laddering pattern, in which a sharply defined series of digestion products spanned from the 1-mers produced upon limit digestion up to 8-mers and beyond (Figure 5C, right). This effect has been observed in two independent HIRA-LacI transfectants (not shown).

The diminished heterogeneity of products of partial digestion of chromatin from DT40 PolyLacO-λ HIRA-LacI cells could reflect more ordered nucleosome phasing. Alternatively, increased nucleosome density may contribute to size homogeneity of partial digestion products. As diagrammed (Figure 5D), a decrease in linker length would limit nuclease access, and cause products of partial digestion to be less heterogeneous in size, thus generating the homogeneous, sharply-defined fragments evident in HIRA-LacI transfectants. Increased loading of nucleosomes would be consistent with the known role of HIRA as a histone chaperone, as well as with the evidence that tethered HIRA increased local enrichment of H3.3 (Figure 5B).

## Discussion

We have shown that localized changes in donor chromatin structure can accelerate gene conversion. Our experiments showed two different chromatin modifiers, the activation domain of the transcription factor VP16, and the histone chaperone, HIRA, promoted gene conversion when tethered to the ψVλ donor array in DT40 B cells. These results identify epigenetic modification as a powerful mechanism for the regulation of homology-directed repair. Additionally, it provides both positive and negative regulation, as permissive donor chromatin structure can stimulate or repressive chromatin structure inhibit [12] homologous recombination.

The DT40 Ig ψVλ donor array normally carries marks typical of transcriptionally active chromatin, including enrichment of AcH3, AcH4, and H3.3. Tethered VP16 or HIRA increased levels of these marks, while activating recombination but not transcription. This suggests that marks that activate recombination and transcription may be overlapping, reflecting dependence of both processes on similarly relaxed chromatin structure. Consistent with this, marks characteristic of activated chromatin correlate with activation of Ig V, D and J segments for V(D)J recombination, a site-specific recombination process that is developmentally regulated, [32] and accompanied by sterile transcription of the target genes [33], [34], [35], [36]. Moreover, transcriptional regulatory elements can contribute to activation of V(D)J recombination [32], [37]; and retroviral integration favors transcribed regions [38].

Tethered VP16 increased levels of AcH3 and AcH4. That histone acetylation might promote gene conversion was suggested by evidence that Ig gene conversion was stimulated by culture of cells with the histone deacetylase inhibitor trichostatin A (TSA) [39], [40]. However, because TSA functions throughout the genome, its effects could be mediated not only by local changes in chromatin structure but also by promoting expression of trans-acting factors which in turn stimulated gene conversion.

Tethered HIRA modulated nucleosome composition by depositing increased levels of H3.3, and appeared to increase local nucleosome density. How might this result in a more permissive chromatin state for gene conversion? Based upon the relative sensitivities of intact nucleosomes to solvent conditions, Jin and Felsenfeld [41] concluded that H3.3-containing nucleosomes were intrinsically less stable, and likely to be more sensitive to chromatin remodeling complexes. Thus, by altering the fraction of H3.3, HIRA might predispose nucleosomes toward displacement during critical steps of homologous recombination. Tethered HIRA had no effect on local histone acetylation, so deposition of H3.3 by HIRA was not sufficient for additional recruitment of HAT activity and local increase of histone acetylation, in contrast to tethered VP16. HIRA has been associated with senescence-associated heterochromatin foci (SAHFs) [42], [43], but we did not find evidence that tethered HIRA induced a heterochromatic state within ψVλ array. We were unable to detect enrichment of diMe-, triMe-H3K9 or the repressive histone variant macroH2A (data not shown), all of which have been demonstrated to be components of SAHFs [42], [43].

Tethered VP16 or HIRA accelerated clonal diversification rates by promoting gene conversion, but this was accompanied by an increase in the fraction of conversion tracts (37% and 24%, respectively) that carried only a single nucleotide substitution, a category essentially absent (3%) in GFP-LacI transfectants. This may reflect a decrease in the physical length of conversion tracts, which could in turn be due to limiting levels of factors necessary to promote gene conversion at the elevated rates dictated by tethered VP16 or HIRA. Alternatively, as gene conversion must involve some form of invasion of the donor by the recipient DNA, alterations in chromatin structure induced by tethered VP16 or HIRA may affect nucleosome position in a way that limits the physical extent of sequence accessible for transfer. For example, the increase in nucleosome density caused by tethered HIRA may be accompanied by a decrease in the average linker length; and, if linker regions are preferred templates for gene conversion, shortening of those regions may contribute to decreased gene conversion tract length in the HIRA transfectants. Alternatively, nucleosome phasing may be changed, so that even though chromatin is more accessible for the homology search, the regions within the ψV segments that can participate in gene conversion are shorter. Finally, the tethered factors may create chromatin that is more accessible to mismatch repair, so a significant fraction of mutations could in principle be reverted, to create a large class of mutants with only single base changes.

Just as epigenetic regulation can activate chromatin for homologous recombination, the disregulation of chromatin structure may promote recombination leading to loss of heterozygosity characteristic of aging cells and of cancer [44], [45], [46], [47]. These events have important implications for human disease, as recombination between nonallelic low copy repeats has been demonstrated to create DNA microdeletions that are associated with complex genetic traits including mental retardation [48], autism [49], and schizophrenia [50]. That activating marks at donor alleles promote gene conversion further suggests that chromatin structure should be considered in design of donors for *in situ* gene correction. The evidence that the same functional endpoint can be achieved by distinct chromatin marks shows that the cell has available multiple, redundant pathways for effecting local alterations of chromatin structure that regulate homologous recombination.

## Materials and Methods

### Cell culturing, imaging, RT-PCR, quantitation of sIgM loss variants and sequence analysis

Cell culture and transfection were carried out as described [12], [21]. Control experiments showed that the PolyLacO tag does not affect cell proliferation, cell cycle profile or Ig gene diversification. The p3′ss-EGFP and p3′ss-EGFP-VP16 plasmids [15], [51] were provided by Dr. Andrew Belmont (University of Illinois); and pH3.3-FLAG [31] by Dr. Gary Felsenfeld (NIH).

For fluorescence imaging, cells (2×10^5^) were cytospun onto glass slides and fixed with 2% paraformaldehyde for 15 min. To visualize the nucleus, cells were stained with DAPI (Sigma, Saint Louis, MO). Fluorescent images were acquired using the DeltaVision microscopy system (Applied Precision) and processed with softWoRx software (Applied Precision).

For RT-PCR, RNA was harvested from cells using TRIzol Reagent (Invitrogen). Vλ and β-actin transcripts were amplified following dilution of the template, with Vλ primers 5′-GTCAGCAAACCCAGGAGAAAC-3′ and 5′-AATCCACAGTCACTGGGCTG-3′; and β-actin as described [52].

To quantitate sIgM loss variants [12], [20], [21], in brief, sIgM^+^ cells were isolated by flow cytometry followed by limiting dilution cloning, expanded for 4 weeks, then approximately 1×10^6^ cells were stained with anti-chicken IgM-RPE (Southern Biotechnology Associates, Birmingham, AL), and analyzed on a FACScan with CellQuest software (BD Biosciences). For sequence analysis, sIgM^−^ cells were sorted, aliquoted to single wells, Vλ regions amplified and sequenced, and their sequences compared to the ψVλ donors to determine if mutations were templated or nontemplated. Sequences were derived from at least two independently transfected lines. Only unique sequences were included for classification of the mutations.

### Chromatin immunoprecipitation (ChIP)

ChIP was carried out as previously described [12]. For all experiments at least two chromatin preparations from at least two independent stably-transfected lines were analyzed. Figures present one representative experiment in which results from analysis of at least three separate amplifications were used to calculate a standard deviation. Separate amplifications of serial dilutions of template DNA were carried out to establish that the measured product intensities were within the linear range. Enrichment of the experimental amplicon was normalized to enrichment of an internal control amplicon from the ovalbumin (Ova) gene, which is not transcribed in chicken B cells. Ova was amplified in the same tube by duplex PCR; and enrichment upon ChIP with specific antibodies was normalized to parallel experiments in which ChIP was carried out with total input DNA controls. Inclusion of the Ova internal control amplicon permitted normalization for IP efficiency, background carryover, differences in effects due to variations in transgene expression, and error due to gel loading. The ψVλ:Ova enrichment ratio from immunoprecipitations was normalized to ψVλ:Ova ratio from total input DNA to determine fold enrichment = [(ψVλ/Ova)Ab]/[(ψVλ/Ova)Input]. As an additional control, the ratio of the experimental and control amplicons in the total input control was compared to a control ChIP with polyspecific IgG; in all cases, enrichment in input and IgG controls were essentially equal.

Antibodies used were: anti H3 CT-pan, anti-AcH3 (06–599), anti-AcH4 (06–866), anti-dimethylated H3(K9) (07–521), dimethylated H3(K9) (07–030), anti-panH3 (05–928), from Upstate (Lake Placid, NY), trimethyl H3(K9) (ab8898; Abcam), and anti-FLAG M2-agarose (A2220; Sigma).

PCR primers for ChIP were:

Vλ_R_: 5′-GCCGTCACTGATTGCCGTTTTCTCCCCTC-3′ and 5′-CGAGACGAGGTCAGCGACTCACCTAGGAC-3′; region between ψVλ1 and Vλ: 5′-CTGTGGCCTGTCAGTGCTTA-3′ and 5′-GCAGGGAACCACAAGAACAT-3′; ψVλ1: 5′-GGGACTTGTGTCACCAGGAT-3′ and 5′-CGCAGTCACATGTGGAATATC-3′; ψVλ5: 5′-GAGCCCCATTTTCTCTCCTC-3′ and 5′-GAGATGTGCAGCAACAAGGA-3′; ψVλ13: 5′-CCCTCTCCCTATGCAGGTTC-3′ and 5′-CCCCTATCACCATACCAGGA-3′; ψVλ18: 5′-CCATTTTCTCCCCTCTCTCC-3′ and 5′-TCACCCTACAGCTTCAGTGC-3′; ψVλ24: 5′-CCATTTTCTCCCCTCTCTCC-3′ and 5′-CAGCCCATCACTCCCTCTTA-3′; ψVλ25: 5′-TCTGTTGGTTTCAGCACAGC-3′ and 5′-GCAGTTCTGTGGGATGAGGT-3′; ψVλ upstream flank: 5′-GGCTCCTGTAGCTGATCCTG-3′ and 5′-GTTCTTTGCTCTTCGGTTGC-3′; ψVλ17 at the PolyLacO-targeted allele: 5′-TAGATAGGGATAACAGGGTAATAGC-3′ and 5′-AGGGCTGTACCTCAGTTTCAC-3′; OVA: 5′-ATTGCGCATTGTTATCCACA-3′ and 5′-TAAGCCCTGCCAGTTCTCAT-3′.

### Micrococcal nuclease digestion and Southern blotting

Nuclei were prepared and digests were performed with 0, 3, 7, 15, 30, and 60 units of MNase as described [53]. Following MNase digestion, DNA was extracted three times with phenol∶chloroform∶isoamyl alcohol, once with chloroform, and precipitated with ethanol; and 20 µg resolved by agarose gel electrophoresis, and transferred for hybridization. The lacO probe was labeled by random priming, using as template a ∼300 bp fragment containing LacO repeats.

## Supporting Information

Figure S1 Sequences of Mutated V Regions from Single DT40 PolyLacO-λ GFP-LacI-VP16 Cells V regions were amplified from single sIgM- cells and then sequenced. Clear blue boxes outline gene conversion events with two or more base changes; blue-shaded boxes outline gene conversion events with one base change; red circles denote point mutations; black dotted boxes indicate insertions; carats denote deletions.
(0.06 MB DOC)

Figure S2 Sequences of Mutated V Regions from Single DT40 PolyLacO-λ HIRA-LacI cells. Notations as in Figure S1.
(0.09 MB DOC)

## References

[1] Paques F, Haber JE (1999). Multiple pathways of recombination induced by double-strand breaks in Saccharomyces cerevisiae.. Microbiol Mol Biol Rev.

[2] West SC (2003). Molecular views of recombination proteins and their control.. Nat Rev Mol Cell Biol.

[3] Bailey JA, Gu Z, Clark RA, Reinert K, Samonte RV (2002). Recent segmental duplications in the human genome.. Science.

[4] Tuzun E, Sharp AJ, Bailey JA, Kaul R, Morrison VA (2005). Fine-scale structural variation of the human genome.. Nat Genet.

[5] Bailey JA, Eichler EE (2006). Primate segmental duplications: crucibles of evolution, diversity and disease.. Nat Rev Genet.

[6] Lupski JR (2007). Genomic rearrangements and sporadic disease.. Nat Genet.

[7] Kaye JA, Melo JA, Cheung SK, Vaze MB, Haber JE (2004). DNA breaks promote genomic instability by impeding proper chromosome segregation.. Curr Biol.

[8] Lobachev K, Vitriol E, Stemple J, Resnick MA, Bloom K (2004). Chromosome fragmentation after induction of a double-strand break is an active process prevented by the RMX repair complex.. Curr Biol.

[9] Shroff R, Arbel-Eden A, Pilch D, Ira G, Bonner WM (2004). Distribution and dynamics of chromatin modification induced by a defined DNA double-strand break.. Curr Biol.

[10] Unal E, Arbel-Eden A, Sattler U, Shroff R, Lichten M (2004). DNA damage response pathway uses histone modification to assemble a double-strand break-specific cohesin domain.. Mol Cell.

[11] Rodrigue A, Lafrance M, Gauthier MC, McDonald D, Hendzel M (2006). Interplay between human DNA repair proteins at a unique double-strand break in vivo.. Embo J.

[12] Cummings WJ, Yabuki M, Ordinario EC, Bednarski DW, Quay S (2007). Chromatin structure regulates gene conversion. PLoS Biology.

[13] Neely KE, Hassan AH, Brown CE, Howe L, Workman JL (2002). Transcription activator interactions with multiple SWI/SNF subunits.. Mol Cell Biol.

[14] Carpenter AE, Memedula S, Plutz MJ, Belmont AS (2005). Common effects of acidic activators on large-scale chromatin structure and transcription.. Mol Cell Biol.

[15] Tumbar T, Sudlow G, Belmont AS (1999). Large-scale chromatin unfolding and remodeling induced by VP16 acidic activation domain.. J Cell Biol.

[16] Memedula S, Belmont AS (2003). Sequential recruitment of HAT and SWI/SNF components to condensed chromatin by VP16.. Curr Biol.

[17] Ray-Gallet D, Quivy JP, Scamps C, Martini EM, Lipinski M (2002). HIRA is critical for a nucleosome assembly pathway independent of DNA synthesis.. Mol Cell.

[18] De Koning L, Corpet A, Haber JE, Almouzni G (2007). Histone chaperones: an escort network regulating histone traffic.. Nat Struct Mol Biol.

[19] Tagami H, Ray-Gallet D, Almouzni G, Nakatani Y (2004). Histone H3.1 and H3.3 complexes mediate nucleosome assembly pathways dependent or independent of DNA synthesis.. Cell.

[20] Sale JE, Calandrini DM, Takata M, Takeda S, Neuberger MS (2001). Ablation of XRCC2/3 transforms immunoglobulin V gene conversion into somatic hypermutation.. Nature.

[21] Yabuki M, Fujii MM, Maizels N (2005). The MRE11-RAD50-NBS1 complex accelerates somatic hypermutation and gene conversion of immunoglobulin variable regions.. Nat Immunol.

[22] Niedzwiedz W, Mosedale G, Johnson M, Ong CY, Pace P (2004). The Fanconi anaemia gene FANCC promotes homologous recombination and error-prone DNA repair.. Mol Cell.

[23] Hatanaka A, Yamazoe M, Sale JE, Takata M, Yamamoto K (2005). Similar effects of Brca2 truncation and Rad51 paralog deficiency on immunoglobulin V gene diversification in DT40 cells support an early role for Rad51 paralogs in homologous recombination.. Mol Cell Biol.

[24] Yamamoto K, Hirano S, Ishiai M, Morishima K, Kitao H (2005). Fanconi anemia protein FANCD2 promotes immunoglobulin gene conversion and DNA repair through a mechanism related to homologous recombination.. Mol Cell Biol.

[25] McIlwraith MJ, Vaisman A, Liu Y, Fanning E, Woodgate R (2005). Human DNA polymerase eta promotes DNA synthesis from strand invasion intermediates of homologous recombination.. Mol Cell.

[26] Kawamoto T, Araki K, Sonoda E, Yamashita YM, Harada K (2005). Dual roles for DNA polymerase eta in homologous DNA recombination and translesion DNA synthesis.. Mol Cell.

[27] Saribasak H, Saribasak NN, Ipek FM, Ellwart JW, Arakawa H (2006). Uracil DNA glycosylase disruption blocks Ig gene conversion and induces transition mutations.. J Immunol.

[28] Lin W, Hashimoto S, Seo H, Shibata T, Ohta K (2008). Modulation of immunoglobulin gene conversion frequency and distribution by the histone deacetylase HDAC2 in chicken DT40.. Genes Cells.

[29] McKittrick E, Gafken PR, Ahmad K, Henikoff S (2004). Histone H3.3 is enriched in covalent modifications associated with active chromatin.. Proc Natl Acad Sci U S A.

[30] Henikoff S (2008). Nucleosome destabilization in the epigenetic regulation of gene expression.. Nat Rev Genet.

[31] Jin C, Felsenfeld G (2006). Distribution of histone H3.3 in hematopoietic cell lineages.. Proc Natl Acad Sci U S A.

[32] Cobb RM, Oestreich KJ, Osipovich OA, Oltz EM (2006). Accessibility control of V(D)J recombination.. Adv Immunol.

[33] Sen R, Oltz E (2006). Genetic and epigenetic regulation of IgH gene assembly.. Curr Opin Immunol.

[34] Chakraborty T, Chowdhury D, Keyes A, Jani A, Subrahmanyam R (2007). Repeat organization and epigenetic regulation of the DH-Cmu domain of the immunoglobulin heavy-chain gene locus.. Mol Cell.

[35] Matthews AG, Kuo AJ, Ramon-Maiques S, Han S, Champagne KS (2007). RAG2 PHD finger couples histone H3 lysine 4 trimethylation with V(D)J recombination.. Nature.

[36] Murre C (2007). Epigenetics of antigen-receptor gene assembly.. Curr Opin Genet Dev.

[37] Jung D, Giallourakis C, Mostoslavsky R, Alt FW (2006). Mechanism and control of V(D)J recombination at the immunoglobulin heavy chain locus.. Annu Rev Immunol.

[38] Bushman F, Lewinski M, Ciuffi A, Barr S, Leipzig J (2005). Genome-wide analysis of retroviral DNA integration.. Nat Rev Microbiol.

[39] Seo H, Masuoka M, Murofushi H, Takeda S, Shibata T (2005). Rapid generation of specific antibodies by enhanced homologous recombination.. Nat Biotechnol.

[40] Kidd JM, Cooper GM, Donahue WF, Hayden HS, Sampas N (2008). Mapping and sequencing of structural variation from eight human genomes.. Nature.

[41] Jin C, Felsenfeld G (2007). Nucleosome stability mediated by histone variants H3.3 and H2A.Z.. Genes Dev.

[42] Zhang R, Poustovoitov MV, Ye X, Santos HA, Chen W (2005). Formation of MacroH2A-containing senescence-associated heterochromatin foci and senescence driven by ASF1a and HIRA.. Dev Cell.

[43] Zhang R, Chen W, Adams PD (2007). Molecular dissection of formation of senescence-associated heterochromatin foci.. Mol Cell Biol.

[44] McMurray MA, Gottschling DE (2003). An age-induced switch to a hyper-recombinational state.. Science.

[45] Tycko B (2003). Genetic and epigenetic mosaicism in cancer precursor tissues.. Ann N Y Acad Sci.

[46] Preston CR, Flores C, Engels WR (2006). Age-dependent usage of double-strand-break repair pathways.. Curr Biol.

[47] Wiktor-Brown DM, Hendricks CA, Olipitz W, Engelward BP (2006). Age-dependent accumulation of recombinant cells in the mouse pancreas revealed by in situ fluorescence imaging.. Proc Natl Acad Sci U S A.

[48] Sharp AJ, Mefford HC, Li K, Baker C, Skinner C (2008). A recurrent 15q13.3 microdeletion syndrome associated with mental retardation and seizures.. Nat Genet.

[49] Weiss LA, Shen Y, Korn JM, Arking DE, Miller DT (2008). Association between microdeletion and microduplication at 16p11.2 and autism.. N Engl J Med.

[50] Walsh T, McClellan JM, McCarthy SE, Addington AM, Pierce SB (2008). Rare structural variants disrupt multiple genes in neurodevelopmental pathways in schizophrenia.. Science.

[51] Tumbar T, Belmont AS (2001). Interphase movements of a DNA chromosome region modulated by VP16 transcriptional activator.. Nat Cell Biol.

[52] Arakawa H, Hauschild J, Buerstedde JM (2002). Requirement of the Activation-Induced Deaminase (AID) gene for immunoglobulin gene conversion.. Science.

[53] Prioleau MN, Nony P, Simpson M, Felsenfeld G (1999). An insulator element and condensed chromatin region separate the chicken beta-globin locus from an independently regulated erythroid-specific folate receptor gene.. Embo J.

