# Supplementary material for: Genetic Variation Stimulated by Epigenetic Modification
Source: PLoS One. 2008 Dec 30;3(12):e4075. doi: 10.1371/journal.pone.0004075 (PMC2605549; doi:10.1371/journal.pone.0004075)
Supplement: Figure S1 — Sequences of Mutated V Regions from Single DT40 PolyLacO-λ GFP-LacI-VP16 Cells V regions were amplified from single sIgM- cells and then sequenced. Clear blue boxes outline gene conversion events with two or more base changes; blue-shaded boxes outline gene conversion events with one base change; red circles denote point mutations; black dotted boxes indicate insertions; carats denote deletions. (0.06 MB DOC) [file pone.0004075.s001.doc]

**VP16-LacI**

Supplementary Figure 1 Cummings et al.

DT40 TCT CCC TCT CCA GGT TCC CTG GTG CAG GCA GCG CTG ACT CAG CCG GCC TCG GTG TCA GCA AAC CCA

VP16-1 --- --- --- --- --- --- --- --- --- --- --- --- --- --- --- --- --- --- --- --- --- ---

VP16-2 --- --- --- --- --- --- --- --- --- --- --- --- --- --- --- T-- --- --- --- --- --- ---

VP16-3 --- --- --- --- --- --- --- --- --- --- --- --- --- --- --- --- --- --- --- --- --- ---

VP16-4 --- --- --- --- --- --- --- --- --- --- --- --- --- --- --- --- --- --- --- --- --- ---

VP16-5 --- --- --- --- --- --- --- --- --- --- --- --- --- --- --- --- --- --- --- --- --- ---

VP16-6 --- --- --- --- --- --- --- --- --- --- --- --- --- --- --- --- --- --- --- --- --- ---

VP16-7 --- --- --- --- --- --- --- --- --- --- --- --- --- --- --- --- --- --- --- --- --- ---

VP16-8 --- --- --- --- --- --- --- --- --- --- --- --- --- --- --- --- --- --- --- --- --- ---

VP16-9 --- --- --- --- --- --- --- --- --- --- --- --- --- --- --- --- --- --- --- --- --- ---

VP16-10 --- --- --- --- --- --- --- --- --- --- --- --- --- --- --- --- --- --- --- --- --- ---

VP16-11 --- --- --- --- --- --- --- --- --- --- --- --- --- --- --- --- --- --- --- --- --- ---

VP16-12 --- --- --- --- --- --- --- --- --- --- --- --- --- --- --- --- --- --- --- --- --- ---

VP16-13 --- --- --- --- --- --- --- --- --- --- --- --- --- --- --- --- --- --- --- --- --- ---

VP16-14 --- --- --- --- --- --- --- --- --- --- --- --- --- --- --- --- --- --- --- --- --- ---

VP16-15 --- --- --- --- --- --- --- --- --- --- --- --- --- --- --- --- --- --- --- --- --- ---

VP16-16 --- --- --- --- --- --- --- --- --- --- --- --- --- --- --- --- --- --- --- --- --- ---

VP16-17 --- --- --- --- --- --- --- --- --- --- --- --- --- --- --- --- --- --- --- --- --- ---

VP16-18 --- --- --- --- --- --- --- --- --- --- --- --- --- --- A-- --- --- --- --- --- ------

VP16-19 --- --- --- --- --- --- --- --- --- --- --- --- --- --- --- --- --- --- --- --- --- ---

VP16-20 --- --- --- --- --- --- --- --- --- --- --- --- --- --- --- --- --- --- --- --- --- ---

VP16-21 --- --- --- --- --- --- --- --- --- --- --- --- --- --- --- --- --- --- --- --- --- ---

VP16-22 --- --- --- --- --- --- --- --- --- --- --- --- --- --- --- --- --- --- --- --- --- ---

VP16-23 --- --- --- --- --- --- --- --- --- --- --- --- --- --- --- --- --- --- --- --- --- ---

VP16-24 --- --- --- --- --- --- --- --- --- --- --- --- --- --- --- --- --- --- --- --- --- ---

VP16-25 --- --- --- --- --- --- --- --- --- --- --- --- --- --- --- --- --- --- --- --- --- ---

DT40 GGA GAA ACC GTC AAG ATC ACC TGC TCC GGG GGT GGC AGC TAT GCT GGA AGT TAC TAT TAT GGC TGG

T

VP16-1 --- --- --- --- --- --- --- --- --- --- --- --- --- --- --- --- --- --- --- --- --- ---

VP16-2 --- --- --- --- --- --- --- --- --- --- --- --- --- --- --- --- --- --- --- --- --- ---

VP16-3 --- --- --- --- --- --- --- --- --- --- --- --- --- --- --- --- --- --- --- --- --- ---

VP16-4 --- --- --- --- --- --- --- --- --- --- --- --- --- --- --- --- --- --- --- --- --- ---

VP16-5 --- --- --- --- --- --- --- --- --- --- --- --- --- --- --- --- --- --- --- --- --- ---

VP16-6 --- --- --- --- --- --- --- --- --- --- --- --- -C- --- --- --- --- --- --- --- --- ---

VP16-7 --- --- --- --- --- --- --- --- --- --- --- --- --- --- --- --- --- --- --- --- --- ---

VP16-8 --- --- --- --- --- --- --- --- --- --- --- --- --- --- --- --- --- --- --- --- --- ---

VP16-9 --- --- --- --- --- --- --- --- --- --- --- --- --- --- --- --- --- --- --- --- --- ---

TGGAAGTTACTATTATG

VP16-10 --- --- --- --- --- --- --- --- --- --- --- --- --- --- --- --- --- --- --- --- --- ---

VP16-11 --- --- --- --- --- --- --- --- --- --- --- --- --- --- --- --- --- --- --- --- --- ---

VP16-12 --- --- --- --- --- --- --- --- --- --- --- --- --- --- --- --- --- --- --- --- --- ---

VP16-13 --- --- --- --- --- --- --- -AT --- --- --- --- --- --- --- --- --- --- --- --- --- ---

TTAC

VP16-14 --- -G- --- --- --- --- --- --- --- --- --- --- --- --- --- --- --- --- --- --- --- ---

VP16-15 --- --- --- --- --- --- --- --- --- --- --- --- -- --- --- --- --- --- --- --- --- ---

VP16-16 --- --- --- --- --- --- --- --- --- --- --- --- --- --- --- --- --- --- --- --- --- ---

G

VP16-17 --- --- --- --- --- --- --- --- --- --- --- --- --- --- --- --- --- --- --- --- --- ---

VP16-18 --- --- --- --- --- --- --- --- --- --- --- --- --- --- --- --- --- --- --- --- --- ---

VP16-19 --- --- --- --- --- --- --- --- --- --- --- --- --- --- --- --- --- --- --- --- --- ---

VP16-20 --- --- --- --- --- --- --- --- --- --- --- --- --- --- --- --- --- --- --- --- --- ---

VP16-21 --- --- --- --- --- --- --- --- --- --- --- --- --- --- --- --- --- --G --- --- --- ---

VP16-22 --- --- --- --- --- --- --- --- --- --- --- --- --- --- --- --- --- --- --- --- --- ---

VP16-23 --- --- --- --- --- --- --- --- --- --- --- --- --- --- --- --- --- --- --- --- --- ---

VP16-24 --- -G- --- --- --- --- --- --- --- --- --- --- --- --- --- --- --- --- --- --- --- ---

VP16-25 --- -G- --- --- --- --- --- --- --- --- -A- -A- --- --- TA- --- --- --- --- --- --- ---

DT40 TAC CAG CAG AAG TCT CCT GGC AGT GCC CCT GTC ACT GTG ATC TAT GAC AAC GAC AAG AGA CCC TCG

VP16-1 --- --- --- --- --- --- --- --- --- --- --- --- --- --- --- --- --- --- --- --- --- ---

VP16-2 --- --- --- --- --- --- --- --- --- --- --- --- --- --- --- --- --- --- --- --- --- ---

VP16-3 --- --- --- --- --- --- --- --- --- --- --- --- --- --- --- --- --- --- --- --- --- ---

VP16-4 --- --- --- --- --- --- --- --- --- --- --- --- --- --- --- --- --- --- --- --- --- ---

VP16-5 --- --- --- --- --- --- --- --- --- --- --- --- --- --- --- --- --- --- --- --- --- ---

VP16-6 --- --- --- --- --- --- --- --- --- --- --- --- --- --- --- --- --- --- --- --- --- ---

VP16-7 --- --- --- --- --- --- --- --- --- --- --- --- --- --- --- --- --- --- --- --- --- ---

VP16-8 --- --- --- --- --- --- --- --- --- --- --- --- --- --- --- --- --- --- --- --- --- ---

VP16-9 --- --- --- --- --- --- --- --- --- --- --- --- --- --- --- --- --- --- --- --- --- ---

VP16-10 --- --- --- --- G-A --- --- --- --- -T- --- --- C-- --- --- T-- --- A-- --- --- --- ---

VP16-11 --- --- --- --- G-A --- --- --- --- -T- --- --- C-- --- --- T-- --- A-- --- --- --- ---

VP16-12 --- --- --- --- G-A --- --- --- --- -T- --- --- C-- --- --- T-- --- A-- --- --- --- ---

VP16-13 --- --- --- --- G-A --- --- --- --- -T- --- --- C-- --- --- T-- --- A-- --- --- --- ---

VP16-14 --- --- --- --- G-A --- --- --- --- -T- --- --- C-- --- --- T-- --- A-- --- --- --- ---

VP16-15 --- --- --- --- G-A --- --- --- --- -T- --- --- C-- --- --- T-- --- A-- --- --- --- ---

VP16-16 --- --- --- --- G-A --- --- --- --- -T- --- --- C-- --- --- T-- --- A-- --- --- --- ---

VP16-17 --- --- --- --- G-A --- --- --- --- -T- --- --- C-- --- --- T-- --- A-- --- --- --- ---

VP16-18 --- --- --- --- G-A --- --- --- --- -T- --- --- C-- --- --- T-- --- A-- --- --- --- ---

VP16-19 --- --- --- --- G-A --- --- --- --- -T- --- --- C-- --- --- --- A-- --- --- --- ---

VP16-20 --G --- --- --- G-A --- --- --- --- -T- --- --- C-- --- --- T-- --- A-- --- --- --- ---

VP16-21 --- --- --- --- G-A --- --- --- --- -T- --- --- C-- --- --- T-- --- A-- --- --- --- ---

VP16-22 --- --- --- --- G-A --- --- --- --- -T- --- --- C-- --- --- T-- --- A-- --- --- --- ---

VP16-23 -T- --- --- --- G-A --- --- --- --- -T- --- --- C-- --- --- T-- --- A-- --- --- --- ---

VP16-24 --- --- --- --- G-A --- --- --- --- -T- --- --- C-- --- --- T-- --- A-- --- --- --- ---

VP16-25 --- --- --- --- --- --- --- --- --- --- --- --- --- --- --- --- --- --- --- --- --- ---

DT40 GAC ATC CCT TCA CGA TTC TCC GGT TCC CTA TCC GGC TCC ACA AAC ACA TTA ACC ATC ACT GGG GTC

VP16-1 --- --- --- --- --- --- --- --- --- --- --- --- --- --- --- --- --- --- --- --- --- ---

VP16-2 --- --- --- --- --- --- --- --- --- --- --- --- --- --- --- --- --- --- --- --- --- ---

VP16-3 --- --- --- --- --- --- --- --- --- --- --- --- --- --- --- --- --- --- --- --- --- ---

VP16-4 --- --- --- --- --- --- --- --- --- --- --- --- --- --- --- --- --- --- --- --- --- ---

VP16-5 --- --- --- --- --- --- --- --- --- --- --- --- --- --- --- --- --- --- --- --- --- ---

VP16-6 --- --- --- --- --- --- --- --- --- --- --- --- --- --- --- --- --- --- --- --- --- ---

VP16-7 A-- --- --- --- --- --- --- --- --- --- --- --- --- --- --- --- --- --- --- --- --- ---

VP16-8 --- --- --- --- --- --- --- --- --- AA- --- --- --- --G GG- --- --- --- --- --- --- ---

VP16-9 --- --- --- --- --- --- --- --- --- --- --- --- --- --- --- --- --- --- --- --- --- ---

VP16-10 --- --- --- --- --- --- --- --- --- AA- --- --- --- --- --- --- --- --- --- --- --- ---

VP16-11 --- --- --- --- --- --- --- --- --- AA- --- --- --- --- --- --- --- --- --- --- --- ---

VP16-12 --- --- --- --- --- --- --- --- --- AA- --- --- --- --- --- --- --- --- --- --- --- ---

VP16-13 --- --- --- --- --- --- --- --- --- AA- --- --- --- --- --- --- --- --- --- --- --- ---

VP16-14 --- --- --- --- --- --- --- --- --- AA- --- --- --- --- --- --- --- --- --- --- --- ---

VP16-15 --- --- --- --- --- --- --- --- --- AA- --- --- --- --- --- --- --- --- --- --- --- ---

VP16-16 --- --- --- --- --- --- --- --- --- --- --- --- --- --- --- --- --- --- --- --- --- ---

VP16-17 --- --- --- --- --- --- --- --- --- AA- --- --- --- --- --- --- --- --- --- --- --- ---

VP16-18 --- --- --- --- --- --- --- --- --- AA- --- --- --- --- --- --- --- --- --- --- --- ---

VP16-19 --- --- --- --- --- --- --- --- --- --- --- --- --- --- --- --- --- --- --- --- --- ---

VP16-20 --- --- --- --- --- --- --- --- --- AA- --- --- --- --- --- --- --- --- --- --- --- ---

VP16-21 --- --- --- --- --- --- --- --- --- AA- --- --- --- --- --- --- --- --- --- --- --- ---

VP16-22 --- --- --- --- --- --- --- --- --- AA- --- --- --- --- --- --- --- --- --- --- --- ---

VP16-23 --- --- --- --- --- --- --- --- --- AA- --- --- --- --- --- --- --- --- --- --- --- ---

VP16-24 --- --- --- --- --- --- --- --- --- AA- --- --- --- --- --- --- --- --- --- --- --- ---

VP16-25 --- --- --- --- --- --- --- --- --- --- --- --- --- --- GC- --- --- --- --- --- --- ---

DT40 CGA GCC GAT GAC GAG GCT GTC TAT TTC TGT GGG AGT GCA GAC AAC AGT GGT GCT GCA TTT GGG GCC

VP16-1 --- --- --- --- --- --- --- --- --- --- --- --- --- --- --- --- --- --- --- --- --- ---

VP16-2 --- --- --- --- --- --- --- --- --- --- --- --- --- --- --- --- --- --- --- --- --- ---

VP16-3 --- --- --- --- --- --- --- --- --- --- --- --- --- --- --- --- --- A-- --- --- --- ---

T

VP16-4 --- --- --- --- --- --- --- --- --- --- --- --- --- --- --- --- --- --- --- --- --- ---

A

VP16-5 --- --- --- --- --- --- --- --- --- --- --- --- --- --- --- --- --- --- --- --- --- ---

VP16-6 --- --- --- --- --- --- --- --- --- --- --- --- --- --- --- --- --- --- --- --- --- ---

VP16-7 --- --- --- --- --- --- --- --- --- --- --- --- --- --- -G- --- --- --- --- --- --- ---

VP16-8 --- --- --- --- --- --- --- --- --- --- --- --- --- --- --- --- --- --- --- --- --- ---

TTGGTGCTA

VP16-9 --- --- --- --- --- --- --- --- --- --- --C --- --- --- --- --- --- --- --- --- --- ---

VP16-10 --- --- --- --- --- --- --- --- --- --- --- --- --- --- --- --- --- --- --- --- --- ---

A

VP16-11 --- --- --- --- --- --- --- --- --- --- --- --- --- --- --- --- --- --- --- --- --- ---

VP16-12 --- --- --- --- --- --- --- --- --- --- --- --- --- --- --- --- --- --- --- --- --- ---

VP16-13 --- --- --- --- --- --- --- --- --- --- --- --- --- --- --- --- --- --- --- --- --- ---

VP16-14 --- --- --- --- --- --- --- --- --- --- --- --- --- --- --- --- --- --- --- --- --- ---

VP16-15 --- --- --- --- --- --- --- --- --- --- --- --- --- --- --- --- --- --- --- --- --- ---

VP16-16 --- --- --- --- --- --- --- --- --- --- --- --- --- --- --- --- --- --- --- --- --- ---

VP16-17 --- --- --- --- --- --- --- --- --- --- --- --- --- --- --- --- --- --- --- --- --- ---

VP16-18 --- --- --- --- --- --- --- --- --- --- --- --- --- --- --- --- --- --- --- --- --- ---

VP16-19 --- --- --- --- --- --- --- --- --- --- --- --- --- --- --- --- --- --- --- --- --- ---

VP16-20 --- --- --- --- --- --- --- --- --- --- --- --- --- --- --- --- --- --- --- --- --- ---

VP16-21 --- --- --- --- --- --- --- --- --- --- --- --- --- --- --- --- --- --- --- --- --- ---

VP16-22 --- --- --- --- --- --- --- --- --- --- --- --- --- --- --- --- -T- --- --- --- --- ---

VP16-23 --- --- --- --- --- --- --- --- --- --- --- --- --- --- --- --- --- --- --- --- --- ---

VP16-24 --- --- --- --- --- --- --- --- --- --- --- --C AT- --- --- --- --- --- --- --- --- ---

TAGTGGTGC

VP16-25 --- --- --- --- --- --- --- --- --- --- --C --- --- --- --- --- --- --- --- --- --- ---

DT40 GGG ACA ACC CTG ACC GTC CTA GGT GAG TCG CTG ACC TCG TCT CGG TCT

VP16-1 --- --- --- --- --- --- --- --- --- --- --- --- --- --- --- ---

VP16-2 --- --- --- --- --- --- --- --- --- --- --- --- --- --- --- ---

VP16-3 --- --- --- --- --- --- --- --- --- --- --- --- --- --- --- ---

VP16-4 --- --- --- --- --- --- --- --- --- --- --- --- --- --- --- ---

VP16-5 --- --- --- --- --- --- --- --- --- --- --- --- --- --- --- ---

VP16-6 --- --- --- --- --- --- --- --- --- --- --- --- --- --- --- ---

VP16-7 --- --- --- --- --- --- --- --- --- --- --- --- --- --- --- ---

VP16-8 --- --- --- --- --- --- --- --- --- --- --- --- --- --- --- ---

VP16-9 --- --- --- --- --- --- --- --- --- --- --- --- --- --- --- ---

VP16-10 --- --- --- --- --- --- --- --- --- --- --- --- --- --- --- ---

VP16-11 --- --- --- --- --- --- --- --- --- --- --- --- --- --- --- ---

VP16-12 --- --- --- --- --- --- --- --- --- --- --- --- --- --- --- ---

VP16-13 --- --- --- --- --- --- --- --- --- --- --- --- --- --- --- ---

VP16-14 --- --- --- --- --- --- --- --- --- --- --- --- --- --- --- ---

VP16-15 --- --- --- --- --- --- --- --- --- --- --- --- --- --- --- ---

VP16-16 --- --- --- --- --- --- --- --- --- --- --- --- --- --- --- ---

VP16-17 --- --- --- --- --- --- --- --- --- --- --- --- --- --- --- ---

VP16-18 --- --- --- --- --- --- --- --- --- --- --- --- --- --- --- ---

VP16-19 --- --- --- --- --- --- --- --- --- --- --- --- --- --- --- ---

VP16-20 --- --- --- --- --- --- --- --- --- --- --- --- --- --- --- ---

VP16-21 --- --- --- --- --- --- --- --- --- --- --- --- --- --- --- ---

VP16-22 --- --- --- --- --- --- --- --- --- --- --- --- --- --- --- ---

VP16-23 --- --- --- --- --- --- --- --- --- --- --- --- --- --- --- ---

VP16-24 --- --- --- --- --- --- --- --- --- --- --- --- --- --- --- ---

VP16-25 --- --- --- --- --- --- --- --- --- --- --- --- --- --- --- ---
