# Supplementary material for: Genetic Variation Stimulated by Epigenetic Modification
Source: PLoS One. 2008 Dec 30;3(12):e4075. doi: 10.1371/journal.pone.0004075 (PMC2605549; doi:10.1371/journal.pone.0004075)
Supplement: Figure S2 — Sequences of Mutated V Regions from Single DT40 PolyLacO-λ HIRA-LacI cells. Notations as in Figure S1. (0.09 MB DOC) [file pone.0004075.s002.doc]

**HIRA-LacI-LacO**

DT40 TCT CCC TCT CCA GGT TCC CTG GTG CAG GCA GCG CTG ACT CAG CCG GCC TCG GTG TCA GCA AAC CTG

HIRA-1 --- --- --- --- --- --- --- --- --- --- --- --- --- --- --- --- --- --- --- --- --- --A

HIRA-2 --- --- --- --- --- --- --- --- --- --- --- --- --- --- --- --- --- --- --- --- --- --A

HIRA-3 --- --- --- --- --- --- --- --- --- --- --- --- --- --- --- --- --- --- --- --- --- --A

HIRA-4 --- --- --- --- --- --- --- --- --- --- --- --- --- --- --- --- --- --- --- --- --- --A

HIRA-5 --- --- --- --- --- --- --- --- --- --- --- --- --- --- --- --- --- --- --- --- --- --A

HIRA-6 --- --- --- --- --- --- --- --- --- --- --- --- --- --- --- --- --- --- --- --- --- --A

HIRA-7 --- --- --- --- --- --- --- --- --- --- --- --- --- --- --- --- --- --- --- --- --- --A

HIRA-8 --- --- --- --- --- --- --- --- --- --- --- --- --- --- --- --- --- --- --- --- --- --A

HIRA-9 --- --- --- --- --- --- --- --- --- --- --- --- --- --- --- --- --- --- --- --- --- --A

HIRA-10 --- --- --- --- --- --- --- --- --- --- --- --- --- --- --- --- --- --- --- --- --- --A

HIRA-11 --- --- --- --- --- --- --- --- --- --- --- --- --- --- --- --- --- --- --- --- --- --A

HIRA-12 --- --- --- --- --- --- --- --- --- --- --- --- --- --- --- --- --- --- --- --- --- --A

HIRA-13 --- --- --- --- --- --- --- --- --- --- --- --- --- --- --- --- --- --- --- --- --- --A

HIRA-14 --- --- --- --- --- --- --- --- --- --- --- --- --- --- --- --- --- --- --- --- --- --A

HIRA-15 --- --- --- --- --- --- --- --- --- --- --- --- --- --- --- --- --- --- --- --- --- --A

HIRA-16 --- --- --- --- --- --- --- --- --- --- --- --- --- --- --- --- --- --- --- --- --- --A

HIRA-17 --- --- --- --- --- --- --- --- --- --- --- --- --- --- --- --- --- --- --- --- --- --A

HIRA-18 --- --- --- --- --- --- --- --- --- --- --- --- --- --- --- T-- --- --- --- --- --- ---

HIRA-19 --- --- --- --- --- --- --- --- --- --- --- --- --- --- --- --- --- --- --- --- --- --A

HIRA-20 --- --- --- --- --- --- --- --- --- --- --- --- --- --- --- --- --- --- --- --- --- --A

HIRA-21 --- --- --- --- --- --- --- --- --- --- --- --- --- --- --- --- --- --- --- --- --- --A

T G

HIRA-22 --- --- --- --- --- --- --- --- --- --- --- --- --- --- --- --- --- --- --- --- --- --A

HIRA-23 --- --- --- --- --- --- --- --- --- --- --- --- --- --- --- --- --- --- --- --- --- --A

G

HIRA-24 GT- --- -G- G-- --- --- --- --- --- --- --- --- --- --- --- --- --- --- --- --- --- --A

HIRA-25 --- --- --- --- --- --- --- --- --- --- --- --- --- --- --- --- --- --- --- --- --- --A

DT40 GGA GGA ACC GTC AAG ATC ACC TGC TCC GGG GGT GGC AGC TAT GCT GGA AGT TAC TAT TAT GGC TGG

HIRA-1 --- -A- --- --- --- --- --- --- --- --- --- --- --- --- --- --- --- --- --- --- --- ---

HIRA-2 --- -A- --- --- --- --- --- --- --- --- --- --- --- --- --- --- --- --- --- --- --- ---

HIRA-3 --- -A- --- --- --- --- --- --- --- --- --- --- --- --- --- --- --- --- --- --- --- ---

HIRA-4 --- -A- --- --- --- --- --- --- --- --- --- --- --- --- --- --- --- --- --- --- --- ---

HIRA-5 --- -A- --- --- --- --- --- --- --- --- --- --- --- --- --- --- --- --- --- --- --- ---

HIRA-6 --- -A- --- --- --- --- --- --- --- --- --- --- --- --- --- --- --- --- --- --- --- ---

HIRA-7 --- -A- --- T-- --- --- --- --- --- --- --- --- --- --- --- --- --- --- --- --- --- ---

HIRA-8 --- -A- --- --- --- --- --- --- --- --- --- --- --- --- --- --- --- --- --- --- --- ---

HIRA-9 --- -A- --- --- --- --- --- --- --- --- --- --- --- --- --- --- --- --- --- --- --- ---

HIRA-10 --- -A- --- --- --- --- --- --- --- --- --- --- --- --- --- --- --- --- --- --- --- ---

HIRA-11 --- -A- --- --- --- --- --- --- --- --- --- --- --- --- --- --- --- --- --- --- --- ---

HIRA-12 --- -A- --- --- --- --- --- --- --- --- --- --- --- --- --- --- --- --- --- --- --- ---

HIRA-13 --- -A- --- --- --- --- --- --- --- --- --- --- --- --- --- --- --- --- --- --- --- ---

HIRA-14 --- -A- --- --- --- --- --- --- --- --- --- --- --- --- --- --- --- --A --- --- --- ---

HIRA-15 --- -A- --- --- --- --- --- --- --- --- --- --- --- --- --- --- --- --G --- --- --- ---

HIRA-16 --- -A- --- --- --- --- --- --- --- --- --- --- --- --- --- --- --- --G --- --- --- ---

HIRA-17 --- -A- --- --- --- --- --- --- --- --- --- --- --- --- --- T-- --- --- --- --- --- ---

HIRA-18 --- --- --- --- --- --- --- --- --- --- --- --- --- --- --- --- --- --- --- --- --- ---

HIRA-19 --- -A- --- --- --- --- --- --- --- --- --- A-- -A- A-C TA- --- --- --- --- --- --- ---

HIRA-20 --- -A- --- --- --- --- --- --- --- --- --- --- --- --- --- --- --- --- --- --- --- ---

TGG

HIRA-21 --- -A- --- --- --- --- --- --- --- --- --- --- --- --- --- --- --- --- --- --- --- ---

CAG

HIRA-22 --- -A- --- --- --- --- --- --- --- --- --- --- --- --- --- --- --- --- --- --- --- ---

HIRA-23 --- -A- --- --- --- --- --- --- --- --- --- --- --- --- --- --- --- --- --- --- --- AA

HIRA-24 --- -A- --- --- --- --- --- --- --- --- --- --- --- --- --- --- --- --- --- --- --- ---

HIRA-25 --- -A- --- --- --- --- --- --- --- --- --- --- --- --- --- --- --- --- --- --- --- ---

Supplementary Figure 2 Cummings et al.

DT40 TAC CAG CAG AAG TCT CCT GGC AGT GCC CCT GTC ACT GTG ATC TAT GAC AAG GAC AAG AGA CCC TCG

HIRA-1 --- --- --- --- --- --- --- --- --- --- --- --- --- --- --- --- --- --- --- --- --- ---

HIRA-2 --- --- --- --- G-A --- --- --- --- --- --- --- --- --- --- --- --- AC- --C --- --- ---

HIRA-3 --- --- --- --- G-A --- --- --- --- --- --- --- --- --- --- --- --- AC- --C --- --- ---

HIRA-4 --- --- --- --- G-A --- --- --- --- --- --- --- --- --- --- --- --- AC- --C --- --- ---

HIRA-5 --- --- --- --- G-A --- --- --- --- -T- --- --- C-- --- --- T-- --- A-- --- --- --- ---

HIRA-6 --- --- --- --- G-A --- --- --- --- -T- --- --- C-- --- --- T-- --- A-- --- --- --- ---

HIRA-7 --- --- --- --- G-A --- --- --- --- -T- --- --- C-- --- --- T-- --- A-- --- --- --- ---

HIRA-8 --- --- --- --- G-A --- --- --- --- -T- --- --- C-- --- --- T-- --- A-- --- --- --- ---

HIRA-9 --- --- --- --- G-A --- --- --- --- -T- --- --- C-- --- --- T-- --- A-- --- --- --- ---

HIRA-10 --- --- --- --- G-A --- --- --- --- -T- --- --- C-- --- --- T-- --- A-- --- --- --- ---

HIRA-11 --- --- --- --- G-A --- --- --- --- -T- --- --- C-- --- --- T-- --- A-- --- --- --- ---

HIRA-12 --- --- --- --- G-A --- --- --- --- -T- --- -G- C-- --- --- T-- --- A-- --- --- --- ---

HIRA-13 --- --- --- --- G-A --- --- --- --- -T- --- --- C-- --- --- T-- --- A-- --- --- --- ---

HIRA-14 --- --- --- --- G-A --- --- --- --- -T- --- --- C-- --- --- T-- --- A-- --- --- --- ---

HIRA-15 --- --- --- --- G-A --- --- --- --- -T- --- --- C-- --- --- T-- --- A-- --- --- --- ---

HIRA-16 --- --- --A --- G-A --- --- --- --- -T- --- --- C-- --- --- T-- --- A-- --- --- --- ---

HIRA-17 --- --- --- --- G-A --- --- --- --- -T- --- --- C-- --- --- T-- --- A-- --- --- --- ---

HIRA-18 --- --- --- --- G-A --- --- --- --- -T- --- -G- C-- --- --- T-- --- A-- --- --- --- ---

HIRA-19 --- --- --- --- G-A --- --- --- --- -T- --- --- C-- --- --- T-- --- A-- --- --- --- ---

HIRA-20 --- --- --- --- G-A --- --- --- --- -T- --- --- C-- --- --- --- A-- --- --- --- ---

HIRA-21 --- --- --- --- G-A --- --- --- --- -T- --- --- C-- --- --- T-- --- A-- --- --- --- ---

HIRA-22 --- --- --- --- G-A --- --- --- --- -T- --- --- C-- --- --- T-- --- A-- --- --- --- ---

HIRA-23 --A --- --- --- G-A --- --- --- --- -T- --- --- C-- --- --- T-- --- A-- --- --- --- ---

HIRA-24 --- --- --- --- G-A --- --- --- --- -T- --- --- C-- --- --- T-- --- A-- --- --- --- ---

HIRA-25 --- --- --- --- G-A --- --- --- --- -T- --- -G- C-- --- --- T-- --- A-- --- --- --- ---

DT40 GAC ATC CCT TCA CGA TTC TCC GGT TCC CTA TCC GGC TCC ACA AAC ACA TTA ACC ATC ACT GGG GTC

HIRA-1 --- --- --- --- --- --- --- --- --- AA- --- --- --- --G GG- --- --- --- --- --- --- ---

HIRA-2 A-- --- --- --- --- --- --- --- --- --- --- --- --- --- --- --- --- --- --- --- --- ---

HIRA-3 --- --- --- --- --- --- --- --- --- --- --- --- --- --- --- --- --- --- --- --- --- ---

HIRA-4 A-- --- --- --- --- --- --- --- --- --- --- --- --- --- --- --- --- --- --- --- --- ---

HIRA-5 --- --- --- --- --- --- --- --- --- --- --- --- --- --- --- --- --- --- --- --- --- ---

HIRA-6 --- --- --- --- --- --- --- --- --- --- --- --- --- --- --- --- --- --- --- --- --- ---

HIRA-7 --- --- --- --- --- --- --- --- --- --- --- --- --- --- --- --- --- --- --- --- --- ---

HIRA-8 --- --- --- --- --- --- --- --- --- --- --- --- --- --- --- --- --- --- --- --- --- ---

HIRA-9 --- --- --- --- --- --- --- --- --- --- --- --- --- --- GC- --- --- --- --- --- --- ---

HIRA-10 --- --- --- --- --- --- --- --- --- --- --- --- --- --- --- --- --- --- --- --- --- ---

HIRA-11 --- --- --- --- --- --- --- --- --- --- --- --- --- --- --- --- --- --- --- --- --- ---

HIRA-12 --- --- --- --- --- --- --- --- --- --- --- --- --- --- --- --- --- --- --- --- --- ---

HIRA-13 --- --- --- --- --- --- --- --- --- --- --- --- --- --- --- --- --- --- --- --- --- ---

HIRA-14 --- --- --- --- --- --- --- --- --- --- --- --- --- --- --- --- --- --- --- --- --- ---

HIRA-15 --- --- --- --- --- --- --- --- --- --- --- --- --- --- --- --- --- --- --- --- --- ---

HIRA-16 --- --- --- --- --- --- --- --- --- --- --- --- --- --- --- --- --- --- --- --- --- ---

HIRA-17 --- --- --- --- --- --- --- --- --- --- --- --- --- --- --- --- --- --- --- --- --- ---

HIRA-18 --- --- --- --- --- --- --- --- --- --- --- --- --- --- --- --- --- --- --- --- --- ---

HIRA-19 --- --- --- --- --- --- --- --- --- --- --- --- --- --- --- --- --- --- --- --- --- ---

HIRA-20 --- --- --- --- --- --- --- --- --- --- --- --- --- --- --- --- --- --- --- --- --- ---

HIRA-21 --- --- --- --- --- --- --- --- --- --- --- --- --- --- --- --- --- --- --- --- --- ---

HIRA-22 --- --- --- --- --- --- --- --- --- --- --- --- --- --- --- --- --- --- --- --- --- ---

HIRA-23 --- --- --- --- --- --- --- --- --- --- --- --- --- --- --- --- --- --- --- --- --- ---

HIRA-24 --- --- --- --- --- --- --- --- --- --- --- --- --- --- --- --- --- --- --- --- --- ---

HIRA-25 --- --- --- --- --- --- --- --- --- --- --- --- --- --- --- --- --- --- --- --- --- ---

DT40 CGA GCC GAT GAC GAG GCT GTC TAT TTC TGT GGG AGT GCA GAC AAC AGT GGT GCT GCA TTT GGG GCC

HIRA-1 --- --- --- --- --- --- --- --- --- --- --- --- --- --- --- --- --- --- --- --- --- ---

HIRA-2 --- --- --- --- --- --- --- --- --- --- --- --- --- --- --- --- --- --- --- --- --- ---

HIRA-3 --- --- --- --- --- --- --- --- --- --- --- --- --- --- --- --- --- --- --- --- --- ---

HIRA-4 --- --- --- --- --- --- --- --- --- --- -T- --- --- --- --- --- --- --- --- --- --- ---

HIRA-5 --- --- --- --- --- --- --- --- --- -T- --- --- --- --- --- --- --- --- --- --- --- ---

HIRA-6 --- --- --- --- --- --- --- --- --- --- --- --- --- --- --- --- --- --- --- --- --- ---

HIRA-7 --- --- --- --- --- --- --- --- --- --- --- --- --- --- --- --- --- --- --- --- --- ---

HIRA-8 --- --- --- --- --- T-- --- --- --- --- --- --- --- --- --- --- --- --- --- --- --- ---

HIRA-9 --- --- --- --- --- --- --- --- --- --- --- --- --- --- --- --- --- --- --- --- --- ---

HIRA-10 --- --- --- --- --- --- --- --- --- --- --- --- --- --- --- --- --- --- --- --- --- ---

HIRA-11 --- --- --- --- --- --- --- --- --- --- --- -C- --- --- --- --- --- --- --- --- --- ---

HIRA-12 --- --- --- --- --- --- --- --- --- --- --- --- --- --- --- --- --- --- --- --- --- ---

HIRA-13 --- --- --- --- --- --- --- --- --- --- --- --- --- --- --- --- --- --- --- --- --- ---

HIRA-14 --- --- --- --- --- --- --- --- --- --- --- --- --- --- --- --- --- --- --- --- --- ---

HIRA-15 --- --- --- --- --- --- --- --- --- --- --- --- --- --- --- --- --- --- --- --- --- ---

HIRA-16 --- --- --- --- --- --- --- --- --- --- --- --- --- --- --- --- --- --- --- --- --- ---

HIRA-17 --- --- --- --- --- --- --- --- --- --- --- --- -T- --- --- --- --- --- --- --- --- ---

HIRA-18 --- --- --- --- --- --- --- --- --- --- --- --- --- --- --- --- --- --- --- --- --- ---

HIRA-19 --- --- --- --- --- --- --- --- --- --- --- --- --- --- --- --- --- --- --- --- --- ---

HIRA-20 --- --- --- --- --- --- --- --- --- --- --- --- --- --- --- --- --- --- --- --- --- ---

CAGGGAG

HIRA-21 --- --- --- --- --- --- --- --- -C- --- --- --- --- --- --- --- --- -A- --- --- --- ---

HIRA-22 --- --- --- --- --- --- --- --- --- --- --- --- --- --- --- --- --- --- --- --- --- ---

HIRA-23 --- --- --- --- --- --- --- --- --- --- --- --- --- --- --- --- -C- --- --- --- --- ---

HIRA-24 --- --- --- --- --- --- --- --- --- --- --- --- --- --- --- --- --- --- --- --- --- ---

AACCATCACTGGGGTCCGAGCCGATGACGAGGCTGTCTATT

HIRA-25 --- --- --- --- --- --- --- --- --- --- --- --- --- --- --- --- --- --- --- --- --- ---

DT40 GGG ACA ACC CTG ACC GTC CTA GGT GAG TCG CTG ACC TCG TCT CGG TCT

HIRA-1 --- --- --- --- --- --- --- --- --- --- --- --- --- --- --- ---

HIRA-2 --- --- --- --- --- --- --- --- --- --- --- --- --- --- --- ---

HIRA-3 --- --- --- --- --- --- --- --- --- --- --- --- --- --- --- ---

HIRA-4 --- --- --- --- --- --- --- --- --- --- --- --- --- --- --- ---

HIRA-5 --- --- --- --- --- --- --- --- --- --- --- --- --- --- --- ---

HIRA-6 --- --- --- --- --- --- --- --- --- --- --- --- --- --- --- ---

HIRA-7 --- --- --- --- --- --- --- --- --- --- --- --- --- --- --- ---

HIRA-8 --- --- --- --- --- --- --- --- --- --- --- --- --- --- --- ---

HIRA-9 --- --- --- --- --- --- --- --- --- --- --- --- --- --- --- ---

HIRA-10 --T --- --- --- --- --- --- --- --- --- --- --- --- --- --- ---

HIRA-11 --- --- --- --- --- --- --- --- --- --- --- --- --- --- --- ---

HIRA-12 --- --- --- --- --- --- --- --- --- --- --- --- --- --- --- ---

HIRA-13 --- -G- --- --- --- --- --- --- --- --- --- --- --- --- --- ---

HIRA-14 --- --- --- --- --- --- --- --- --- --- --- --- --- --- --- ---

HIRA-15 --- --- --- --- --- --- --- --- --- --- --- --- --- --- --- ---

HIRA-16 --- --- --- --- --- --- --- --- --- --- --- --- --- --- --- ---

HIRA-17 --- --- --- --- --- --- --- --- --- --- --- --- --- --- --- ---

HIRA-18 --- --- --- --- --- --- --- --- --- --- --- --- --- --- --- ---

HIRA-19 --- --- --- --- --- --- --- --- --- --- --- --- --- --- --- ---

HIRA-20 --- --- --- --- --- --- --- --- --- --- --- --- --- --- --- ---

HIRA-21 --- --- --- --- --- --- --- --- --- --- --- --- --- --- --- ---

HIRA-22 --- --- --- --- --- --- --- --- --- --- --- --- --- --- --- ---

HIRA-23 --- --- --- --- --- --- --- --- --- --- --- --- --- --- --- ---

HIRA-24 --- --- --- --- --- --- --- --- --- --- --- --- --- --- --- ---

HIRA-25 --- --- --- --- --- --- --- --- --- --- --- --- --- --- --- ---
